# Recent Progress and Advances in Stimuli-Responsive Polymers for Cancer Therapy

**DOI:** 10.3389/fbioe.2018.00110

**Published:** 2018-08-13

**Authors:** N. Vijayakameswara Rao, Hyewon Ko, Jeongjin Lee, Jae Hyung Park

**Affiliations:** ^1^School of Chemical Engineering, College of Engineering, Sungkyunkwan University, Suwon, South Korea; ^2^Department of Health Sciences and Technology, SAIHST, Sungkyunkwan University, Suwon, South Korea; ^3^Biomedical Institute for Convergence at SKKU, Sungkyunkwan University, Suwon, South Korea

**Keywords:** chemotherapy, pH, redox, hypoxia, ROS, light-triggered polymers, temperature-responsive polymers, cancer therapy

## Abstract

The conventional chemotherapeutic agents, used for cancer chemotherapy, have major limitations including non-specificity, ubiquitous biodistribution, low concentration in tumor tissue, and systemic toxicity. In recent years, owing to their unique features, polymeric nanoparticles have been widely used for the target-specific delivery of drugs in the body. Although polymeric nanoparticles have addressed a number of important issues, the bioavailability of drugs at the disease site, and especially upon cellular internalization, remains a challenge. A polymer nanocarrier system with a stimuli-responsive property (e.g., pH, temperature, or redox potential), for example, would be amenable to address the intracellular delivery barriers by taking advantage of pH, temperature, or redox potentials. With a greater understanding of the difference between normal and pathological tissues, there is a highly promising role of stimuli-responsive nanocarriers for drug delivery in the future. In this review, we highlighted the recent advances in different types of stimuli-responsive polymers for drug delivery.

## Introduction

Cancer is a common cause of death every year worldwide (Siegel et al., 2017). Chemotherapy is the major treatment for cancer patients. Nevertheless, the major limitation to the clinical application of these drugs is their short half-life and wide bio-distribution (Praga et al., 1980). The significant drawbacks of conventional drug delivery approaches are non-specific bio-distribution and low selectivity. As a result, normal cells are also exposed to the cytotoxic effects of these drugs. Actually, in many cases, only a small portion of the administered drug reaches the tumor site (Muller and Keck, 2004). Many chemotherapeutic drugs have long-term side effects in the heart, lungs, and kidneys (Pérez-Herrero and Fernández-Medarde, 2015). As a result, chemotherapeutic drugs can cause side effects such as nausea, vomiting, immune suppression, hepatotoxicity, nephrotoxicity, memory loss, anemia, and even death. It is therefore essential to develop new drug delivery systems to overcome these limitations and to improve the efficacy of cancer treatments (Kataoka et al., 2005). To overcome these limitations, nanotechnology has been extensively studied for potential applications in cancer diagnosis and treatment including liposomes, dendrimers, polymeric nanoparticles, and lipoprotein drug carriers, among others. Among many nanotechnology approaches, polymer nanoparticles have gained significant attention as a nanomedicine platform in the field of drug delivery (Langer and Folkman, 1976; Duncan and Kopeček, 1984; Duncan, 2003, 2006; Brannon-Peppas and Blanchette, 2004; Torchilin, 2007; Yongfeng et al., 2010). Although polymers have been reported to preferentially accumulate in tumors due to passive targeting and receptor-mediated active targeting, the traditional polymers are accompanied by systemic adverse effects that are mostly attributable to their non-specific bio-distribution and uncontrollable drug release (Zulkifli et al., 2017). To overcome these barriers, stimuli-sensitive polymer nanoparticles have been developed to achieve the controlled release of payloads at the target sites. In comparison to the traditional polymer nanocarriers, stimuli-sensitive nanoparticles could successfully lower the dosage frequency, while retaining the drug concentration in targeted organs/tissues for a much longer period. In this aspect, the stimuli-sensitive nanoparticles offer interesting properties for reducing drug concentration fluctuation and drug toxicities and improving therapeutic efficacy (Gil and Hudson, 2004; MacEwan et al., 2010; Taghizadeh et al., 2015). They are considered intelligent, smart, or environmentally-responsive polymers. Remarkable initiatives have been dedicated to the advancement of stimuli-responsive polymers that can successfully deliver therapeutic agents to disease sites. Common stimuli explored by stimuli-responsive polymers include endogenous [e.g., reactive oxygen species (ROS), redox, pH, and enzymes] and exogenous (e.g., light, temperature, magnetic field, and ultrasound) stimuli (Gil and Hudson, 2004; Cheng et al., 2014). Although current research and reviews have established numerous novel aspects for stimuli-responsive nanoplatforms as smart drug carriers (Couvreur, 2013; Hrubý et al., 2015; Lee et al., 2015; Liu et al., 2016; Zhou et al., 2018), few of them have been reached into clinical studies. Due to the absence of a standardized manufacturing method, the toxicity of nanocarriers has prevented them from receiving regulatory and ethical approval. As a result, such stimulus-sensitive nanocarriers are not presently authorized for clinical use. There are numerous important elements that need to be addressed for future development of stimuli-responsive nanoplatforms, such as nanocarrier biocompatibility and biodegradability, drug loading capacity, nanocarrier stability, and low toxicity. To date, many stimuli-responsive polymers have been developed for controlled release of drugs. In this review, we summarize different stimuli-responsive polymers reported in the literature in pH-responsive, redox-responsive, temperature-responsive, light, ROS, and hypoxia-sensitive polymers for drug delivery applications.

## Endogenous stimuli-responsive polymers

### pH-responsive polymers

**Figure 1:**
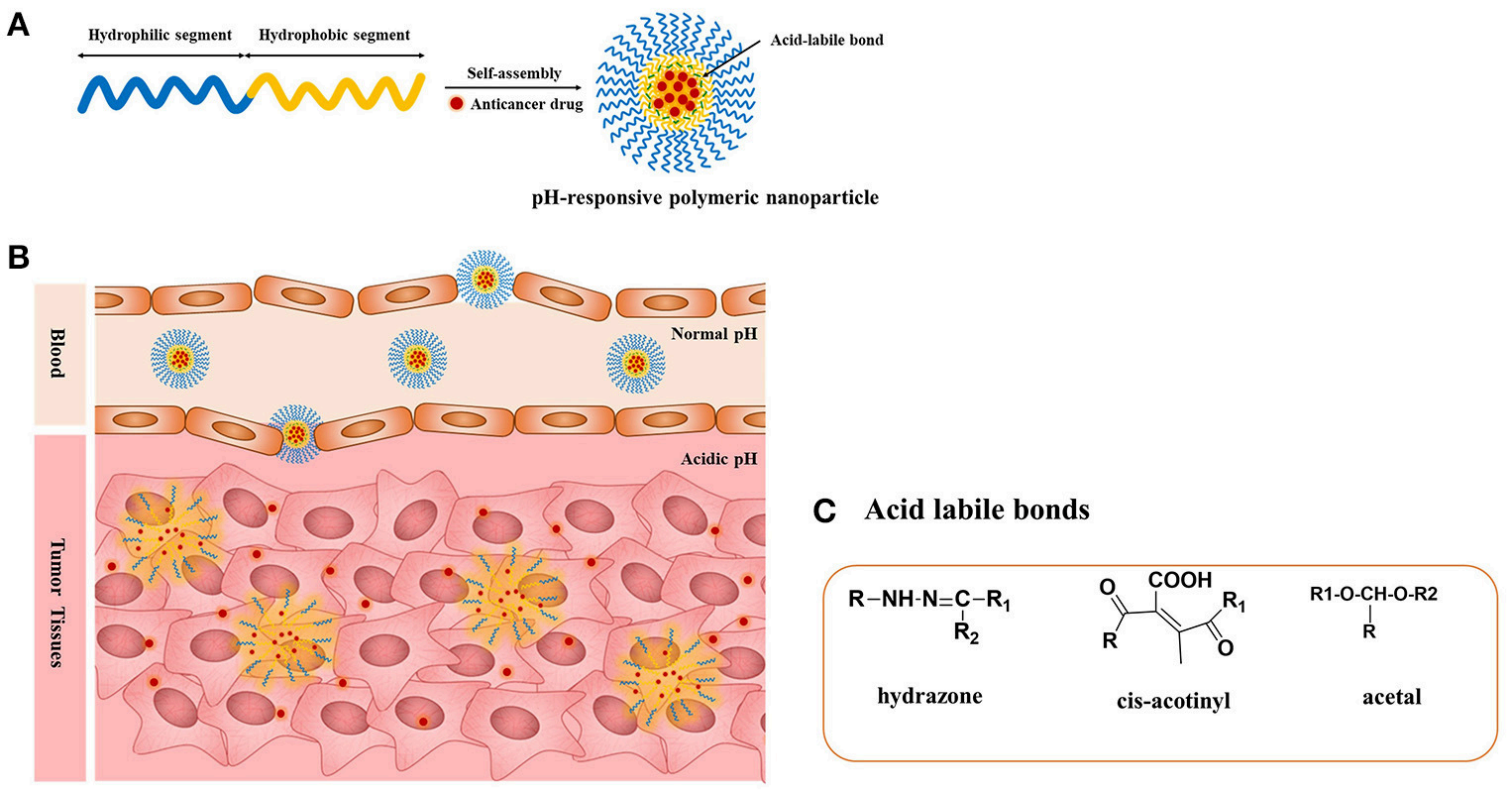
**(A)** pH-responsive copolymers in which the anticancer drugs are conjugated via the acid-liable bonds; **(B)** pH-sensitive polymeric micelles drug release mechanism under the acidic environment such as solid tumors, endosomes, and lysosomes through the cleavage of the acid-labile bonds; **(C)** chemical structures of acid liable chemical bonds.

The pH-responsive polymer nanoparticles have gained academic and commercial interest in the last two decades for applications in cancer diagnosis and therapy. In cancer therapy, the tumor microenvironment is considered an ideal trigger for the selective release of anticancer drugs in tumor tissues and within tumor cells (Duncan, 2003; Cabane et al., 2012; Liu et al., 2013; Mura et al., 2013; Chang et al., 2016). The extracellular regions around normal tissues and blood have a constant pH of 7.4, but the extracellular pH region of tumors ranges from 6.0 to 6.5 (Duncan and Kopeček, 1984; Rao et al., 2016; Kocak et al., 2017). This distinction in pH between normal and tumor tissues in endosomal and lysosomal compartments can be used as an internal stimulus for triggered drug release for chemotherapy (Bae et al., 2003; Cairns et al., 2011; Liu et al., 2013). By choosing the best material composition, it is feasible to engineer nanocarriers that could make use of these pH differences and allow for delivery of the encapsulated payload to select extracellular or intracellular sites (Ulbrich and Šubr, 2004). The pH-sensitive polymeric micelles have been used for targeting drug delivery to tumors because they are stable at physiological pH; in addition, such micelles are deformed to assist the release of the drug under mildly acidic conditions outside or inside tumor cells, which could enhance therapeutic efficacy and reduce side effects (Figure 1, Table 1). The pH-sensitive polymeric micelles could be used to control the release of hydrophobic agents in tumor tissue through the enhanced permeability and retention (EPR) effect and depending on the low pH of tumor tissue. The drug release from micelles at the targeted tumor area could be improved by applying an internal or external trigger. These systems enhance the accumulation at tumor sites or intracellular compartments in tumor cells with less drug distribution and therefore decrease damage to healthy tissues.

**Table 1:** Extracellular and intracellular microenvironments targeted drug delivery.

**Polymeric micelles**	**Drug**	**Results**	**References**
N-Boc-histidine-poly[(D,L-lactide)]-co-glycolide]- poly(ethylene glycol)-poly[(D,L-lactide)]-coglycolide] micelles	Doxorubicin	Exhibit excellent cellular uptake. This nanocarrier is good carrier in view of its biodegradability, biocompatibility, and sensitivity to tumor extracellular pH.	Chang et al., 2010
Poly(ethylene glycol)-cis-aconityl-chitosan-stearic acid polymeric micelles	Doxorubicin	Efficient internalization to tumor cells. Furthermore, *in vivo* studies indicated that better antitumor activity on xenograft tumor model.	Hu et al., 2012
Poly[(D,L-lactide)]-co-glycolide]-poly(ethylene glycol)- folate (PLGA-PEG-FOL) and poly (b-amino ester)- poly(ethylene glycol)-folate (PAE-PEG-FOL) mixed micelles	Doxorubicin	The micelles exhibited a higher degree of cellular uptake due to folate receptor-mediated endocytosis, and exhibit higher cytotoxicity due to trigger drug release at endosomal pH.	Zhao et al., 2010
poly(ethylene glycol)-poly(D,L-lactic acid)-poly (β amino ester) [PEG-(PLA-PAE)] micelles	Doxorubicin	The release of doxorubicin from the micelles was accelerated by decreasing pH from 7.4 to 5.0.	Zhang et al., 2012
Poly(ethylene glycol)-poly(L-histidine)/poly(ethylene glycol)-poly(L-lactic acid) [PEG-PHis/PEG-PLA] mixed micelles	Doxorubicin	Efficient internalization to tumor cells. The *in vitro* results indicated that the micelles were more effective in tumor cell kill because of accelerated drug release and folate receptor-mediated tumor uptake.	Lee et al., 2003
Poly(HEMA-co-histidine)-poly(D, L-lactic acid) and folate-poly(ethylene glycol)-poly(D, L-lactic acid) mixed micelles	Doxorubicin	The micelles exhibited great anti-tumor efficacy with folate mediated cancer targeting and pH triggered intracellular drug delivery. *In vivo* results revealed that specific targeting of folate–micelles exhibited cancer targeting. These multifunctional micelles have great scope in cancer diagnosis and therapy.	Tsai et al., 2010
Poly(ethylene glycol)-poly(mono-2,4,6-trimethoxy benzylidene-pentaerythritol carbonate) [PEG-b- P(TMBPEC-co-AC)] micelles	Paclitaxel	The micelles showed high anti-tumor activity with superior extracellular stability and rapid intracellular drug release.	Wu et al., 2012

The pH-responsive polymers are developed by two strategies. The most important strategy for pH-sensitive drug release is to use acid-labile linkers to conjugate drugs covalently to carrier molecules or to the surfaces of nanostructures, forming prodrugs that are inactive until the linker is hydrolyzed. The presence of acid-sensitive spacers between the polymer and drug facilitates drug release either in acidic extracellular fluids or after endocytosis in endosomes or lysosomes of tumor cells. A broad spectrum of cleavable linkers has been used in the field of acid-sensitive drug delivery, including hydrazine (Bae et al., 2003), acetal (Gillies and Frechet, 2003), benzoic imine (Chao et al., 2011), and ortho-ester (Yang et al., 2012). The other strategy is use of a class of polyelectrolytes with ionizable groups. By changing the environmental pH or the ionic composition, smart polyelectrolytes are ionized and can dramatically change their conformation for drug release. In addition to the polyelectrolytes, a variety of self-assembled or novel polymeric structures have been developed for controlled drug delivery, such as micelles. In this review, we summarize the application of pH-sensitive polymeric nanocarriers including pH-sensitive micelles for tumor chemotherapy.

Chang et al. reported a polymer micelle consisting of poly[(D,L-lactide)]-co-glycolide]-PEG-poly[(D,L-lactide)] coglycolide] copolymer capped with N-Boc-histidine (Chang et al., 2010). This nanocarrier showed good biodegradability and biocompatibility after successful modification with N-Boc-histidine. The anticancer drug was loaded into micelles that were utilized for trigged DOX drug release in extracellular tumor microenvironments. The DOX release was higher at pH 6.2 compared to pH 7.4. The cellular uptake of DOX was carried out in human breast cancer cells. Greater uptake of DOX was observed at pH 6.2 as result of DOX rapid release at tumor pH. Thus, the pH-induced release of drug after accumulation of the micelles in the tumor sites through enhanced permeability offered a more efficient method of chemotherapy by providing a higher local concentration of the drug at tumor sites and minimal release of the drug from micelles throughout blood circulation (pH 7.4). The above example illustrates that tumor pH is a good method of tumor targeting. Nevertheless, although drug release from the pH-sensitive micelles triggered by the tumor pH could substantially reduce the systemic toxicity and also enhance *in vitro* and *in vivo* anticancer activity, it could not solve the problem of multidrug resistance of tumor cells to anticancer drugs. To attain the maximum pharmacological effect and to improve the therapeutic effect of anticancer drugs, the antitumor drugs should be released rapidly from the micelles in the acidic microenvironment of endosomes/lysosomes. In addition, the use of drug-loaded micelles that destabilize at an early endosomal pH of 6.0 should maximize intracellular drug delivery and minimize drug release at the extracellular pH and at the lysosomal pH. In another report, Hu et al. reported PEG-cis-aconityl-chitosan-stearic acid polymeric micelles for trigged DOX drug release (Hu et al., 2012). PEGylation reduced cytotoxicity of the conjugate. The acid-triggered PEG degradation combined with DOX release was attributed to high internalization in tumor cells. The cytotoxicity of micelles was higher compared to that of free DOX. In a recent work, Bae et al. prepared intracellular pH-sensitive polymeric micelles of PEG-poly(aspartate-hydrazone-adriamycin) that can release the drug DOX at endosomes (pH 5.0–6.0) and lysosomes (pH 4.0–5.0). This designed polymer maximizes the DOX delivery efficiency to the tumor tissue (Bae et al., 2005). The micelles exhibited proper intracellular pH-triggered drug release capability and effective antitumor suppression with low toxicity. In another recent report, Wu et al. reported monoclonal antibody 2C5-DSPE-poly(ethylene glycol) and poly (histidine)-poly(ethylene glycol) mixed micelles for paclitaxel drug delivery (Wu et al., 2013). These micelles enhanced tumor internalization by 2C5-mediated endocytosis and triggered drug release, resulting in improved anticancer efficacy. In another report, Zhang et al. prepared PEG-poly(D, L-lactic acid)-poly (amino ester) [PEG-(PLA-PAE)] micelles. Interestingly, the release of DOX from the micelles was observed by decreasing pH from 7.4 to 5.0 (Zhang et al., 2012). In another report, Wu et al. prepared PEG-poly(mono-2,4,6-trimethoxy-benzylidene-pentaerythritol carbonate) [PEG-b-P(TMBPEC-co-AC)] micelles for paclitaxel intracellular drug release (Wu et al., 2012). These micelles had high anti-tumor activity with good extracellular stability. The polymeric micelles combined with active targeting and pH sensitivity as a triggered drug release mechanism offer challenging opportunities to improve therapeutic efficacy. Bae et al. synthesized pH-sensitive multifunctional micelles anchored with biotin that can bind its receptors only if subjected to an acidic tumor extracellular environment. At neutral pH, biotin was protected by a PEG shell of the corona and therefore not available for binding. When the micelles were exposed to the acidic extracellular fluid of tumors, the micelle destabilized, causing enhanced drug release and disrupted endosomal membrane (Lee et al., 2008). A pH-sensitive poly(histidine)-PEG/DSPE-PEG micelle was prepared for quick intracellular drug release in response to the acidity in endosomes. In this way, higher intracellular drug concentrations are obtained. Tasi et al. reported poly(HEMA-co-histidine)-poly(D, L-lactic acid) and folate-poly(ethylene glycol)-poly(D, L-lactic acid) mixed micelles prepared for pH-triggered intracellular DOX drug delivery (Tsai et al., 2010). These micelles exhibited high anti-tumor efficacy with folate-mediated cancer targeting. In another report, Zhao et al. prepared mixed micelles consisting of poly[(D, L-lactide)]-co-glycolide]-PEG- folate (PLGA-PEG-FOL) and poly (β-amino ester)-poly(ethylene glycol)-folate (PAE-PEG-FOL) for endosomal pH-triggered DOX release (Zhao et al., 2010). These polymer micelles showed improved cytotoxicity. As shown in these studies, when the ligands conjugated to the micelles bind to their specific receptors on the cell membrane, the micelles are be internalized by endocytosis, and the combination of active targeting and triggered release resulted in superior cytotoxicity and antitumor activity as compared to non-multifunctional micelles. When intracellular pH-triggered drug release is integrated with receptor-mediated active targeting, the multifunctional polymeric micelles have advantages: (1) polymeric micelles can selectively recognize cancer cells via receptor-mediated binding (2) and can release the drug at tumor sites. This combination could help to inhibit tumor growth and reverse multidrug resistance (MDR). Lee et al. prepared DOX-loaded PEG-poly(L-histidine)/PEG-poly(L-lactic acid) mixed micelles to challenge MDR in cancers (Lee et al., 2003). The micelles showed MDR reversing via receptor-mediated endocytosis. Poly (histidine)-b-PEG-b-PLLA-based pH-sensitive micelles decorated with folic acid were prepared for active targeting to overcome multidrug resistance. When the DOX-loaded poly(histidine)-b-PEG-b-PLLA micelles were internalized into tumor cells via folate receptor-mediated active endocytosis, the pH-responsive poly(histidine) block became protonated in the endosomes, causing micelle destabilization and release of DOX into the cytosol by disruption of the endosomal membrane, which could efficiently kill both DOX-sensitive and resistant tumor cells through a high dose of DOX in the cytosol.

**Figure 2:**
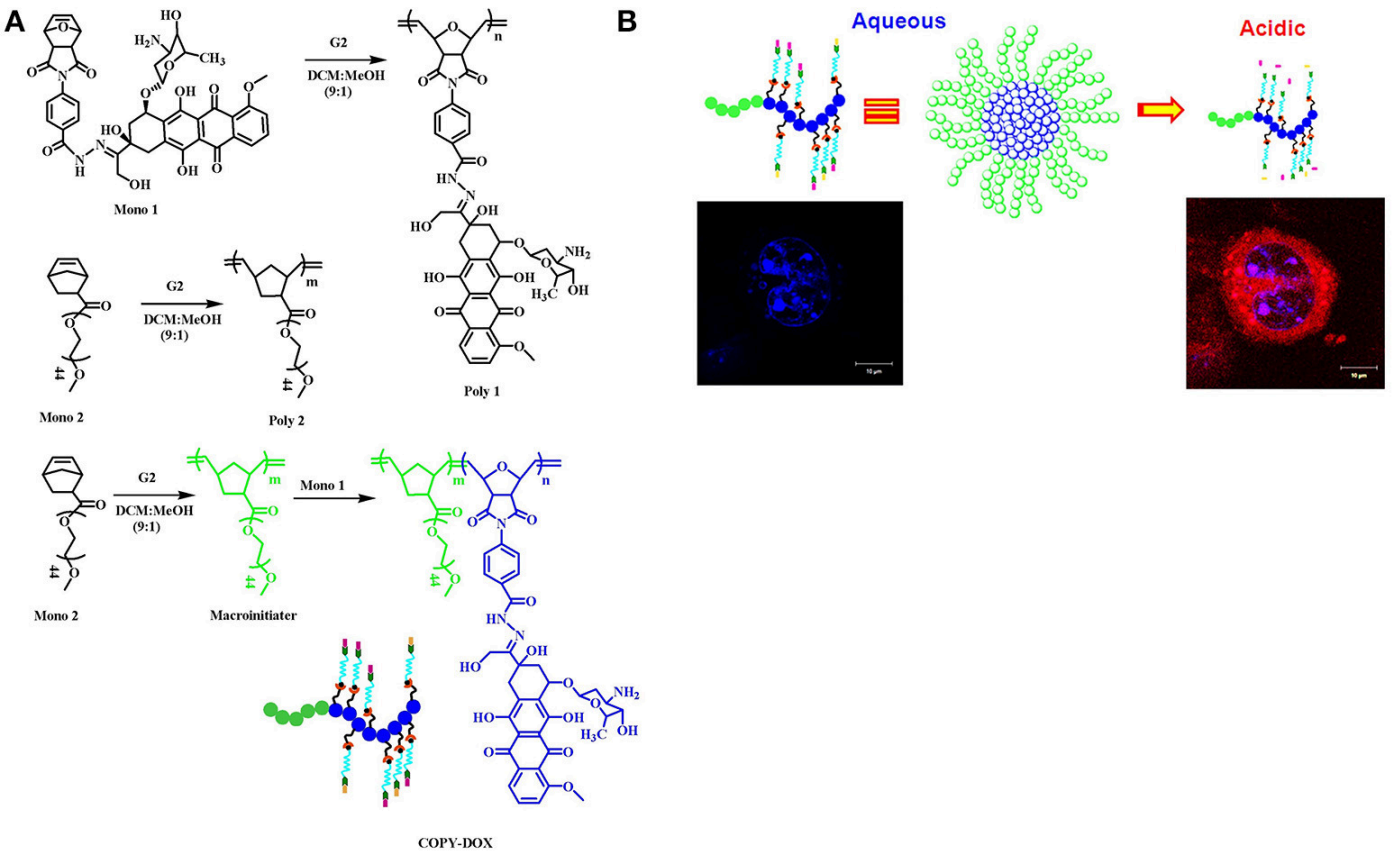
Norbornene-derived doxorubicin copolymers as drug carriers with pH-responsive hydrazone linker. **(A)** Synthesis of block copolymers. **(B)** A cartoon representation of breaking of hydrazone linkage at acidic pH and releasing the drug reprinted from Rao et al. (2012) with the permission of ACS publications.

**Figure 3:**
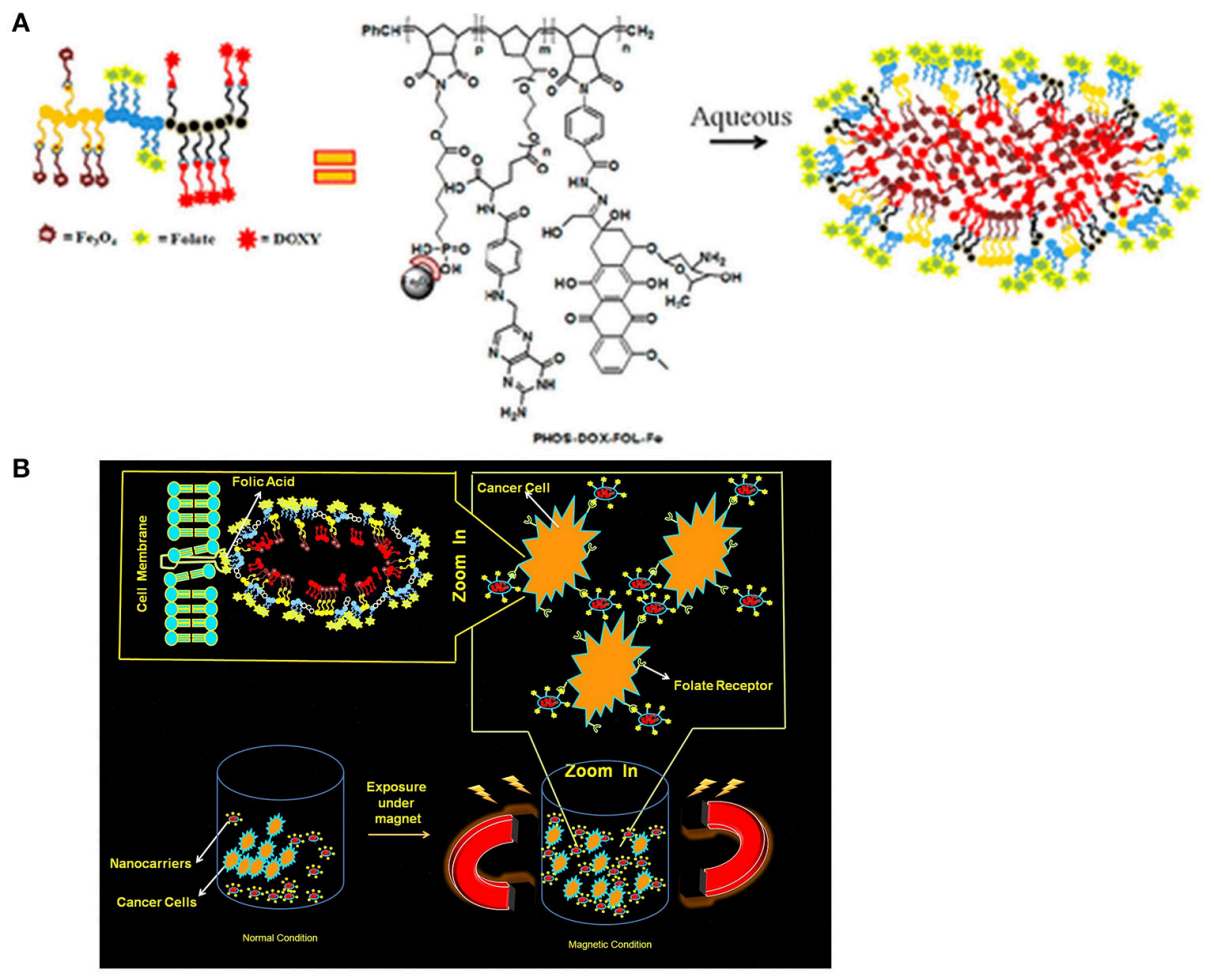
Magnetic norbornene polymer as a multi-responsive nanocarrier for site-specific cancer therapy reprinted by Rao et al. (2014b) with the permission of ACS publications. **(A)** Cartoon representation of self-assembly. **(B)** The cartoon representation of the magnetic field induced and receptor-mediated endocytosis of triblock copolymer.

The pH-sensitive polymeric carrier poly(vinylpyrrolidone-co-dimethyl maleic anhydride) (PVD) was attached to doxorubicin (DOX) via an acid-sensitive linker that could release free drug under mild acidic conditions (Kamada et al., 2004). The results indicated that the polymer carrier exhibited high anticancer activity and enhanced accumulation of drug due to controlled release. Ulbrich et al. have prepared the antibody-targeted pH-sensitive polymer-DOX nanocarrier. The DOX was attached to a polymer carrier via a hydrazone linker. Hydrazone linkage hydrolytically controls the release of DOX from the carrier and its activation after transfer of the polymer-drug from the blood circulation and extracellular environment into intracellular compartments. Unlike classic conjugates, these polymer conjugates do not require lysosomal enzymes for biological activity. In another polymer nanocarrier, HPMA copolymers are conjugated to DOX via a pH-sensitive linker. Rao et al. synthesized norbornene copolymers as a DOX carrier with hydrazone linkers (Rao et al., 2012). In the first step, norbornene-derived DOX hydrazone linker (monomer 1) was prepared (Figure 2A). Then, a second monomer norbornene-derived PEG was synthesized (monomer 2). Finally, copolymerization was performed using ring-opening metathesis polymerization to prepare the block copolymer. The drug release behavior was observed under mildly acidic conditions resembling the pH of cancerous cells (Figure 2B). The copolymer exhibited high anticancer efficacy against HeLa and 4T cancer cells. In another report, Rao et al. synthesized stimuli-responsive norbornene triblock copolymers consisting of diethoxyphosphoryl)hexanoate (PHOS), PEG-folate, and doxorubicin (Rao et al., 2014b). The PHOS groups were used to anchor iron particles (Figure 3A). The drug-release profile indicated cleavage of the hydrazone linker at mildly acidic conditions resembling the pH of cancerous cells. The nanocarrier showed greater internalization because of the magnetic field (Figure 3B). This nanocarrier exhibited high intracellular DOX release because of folate (FOL) receptor. In another recent report, Rao et al. synthesized multiple pH-responsive chemotherapeutic agent nanocarriers by conjugating doxorubicin, indomethacin, and folate to the backbone of norbornene polymer (V R et al., 2014). This nanocarrier showed more anticancer activity due to the multidrug delivery and presence of FOL receptor enables the nanocarrier to act as dual sensitive tumor targeting. The self-assembled nanocarrier delivered drugs in mildly acidic conditions. In another report, Rao et al. reported norbornene-based triblock copolymers for sustained release of multi-cancer drugs using an ester linker (Rao et al., 2014a). The drug-release data indicated cleavage of the ester linker at mildly acidic conditions resembling the pH of cancerous cells. This copolymer exhibited excellent cellular internalization and good anticancer efficacy. Guo et al. reported folic acid-conjugated poly(ethylene glycol)-poly(ε-caprolactone) copolymer. DOX was further connected with a hydrazone linker for pH-triggered drug release. The polymeric micelles exhibited tumor accumulation, which improved delivery efficiency and cancer-targeting specificity. The hydrazone linker is often used to conjugate polymers to the ketone group in DOX. However, the acid-labile hydrazone linker is unstable *in vivo*, with a half-life in plasma of 48–72 h, less than that of the antibody moiety. In some cases, the hydrazone linker can induce cyclic reaction and release less active DOX instead of free DOX. Thambi et al. reported an acid sensitive orthoester linkage composed of poly(ethylene glycol) (PEG) and hydrophobic poly(γ-benzyl L-glutamate) as a carrier to release DOX at mildly acidic conditions (Thambi et al., 2011). *In vitro* release studies demonstrated that DOX is slowly released from PNPs in physiological buffer (pH 7.4), whereas DOX is released significantly more under acidic conditions (pH 5.0). Interestingly, DOX-loaded pH-sensitive PNPs exhibited higher toxicity to SCC7 cancer cells than DOX-loaded micelles without the pH-sensitive linker. Heller et al. prepared biodegradable polyacetal biodegradable carriers, which hydrolyze at acidic pH. Etrych et al. synthesized an HPMA drug carrier with paclitaxel (PTX) and docetaxel (DTX) via the hydrolytically unstable hydrazone linkage (Etrych et al., 2010). The conjugates were stable at the pH of blood (7.4) and released drugs under mildly acidic conditions (pH 5.0) in cancer cells. Overall, the HPMA polymer conjugate was demonstrated to be very potent for effective tumor delivery and treatment. Liu et al. prepared micelles based on mPEG-*b*-poly (aspartate hydrazone doxorubicin), in which DOX was conjugated to hydrophobic segments through an acid-sensitive hydrazone linker (Liu et al., 2013). Selective release of DOX at endosomal pH suppressed tumor growth in mice with enhanced therapeutic efficacy and decreased systemic toxicity compared to free DOX. These results indicated that the hydrazone linkages could be stable at physiological pH 7.4 but degrade effectively at the lower pH of endosomes and lysosomes (pH 5–6) to release the drug. In another recent report, Seung Han et al. reported CMD and docetaxel (DTX) with ester linkers (Seung Han et al., 2015). The polymer conjugate exhibited sustained release of DTX in pH 7.4, and its release rate increased remarkably under mildly acidic conditions resembling the intracellular environment. *In vitro* cytotoxicity data demonstrated that the conjugate exhibited higher toxicity to cancer cells under mildly acidic conditions compared to pH 7.4. The polymer conjugate accumulated efficiently at the tumor site and showed high antitumor efficacy. In another work, Ganivada et al. reported a copolymer using ring-opening polymerization (ROP) and click chemistry for site-specific sustained delivery of the antitumor drug DOX (Ganivada et al., 2016). DOX was attached to the polymer via an oxime linker. The drug-release profile indicated drug release under mildly acidic conditions. From cell viability studies, it was evident that these polymer micelles exhibited high tumor efficacy.

Despite the advantages of pH-responsive polymer nanocarriers such as controlled drug release, high specificity to the tumor, intracellular drug delivery, and excellent therapeutic efficacy with reduced side effects, pH-responsive polymer nanocarriers face severe challenges. Although many studies on pH-responsive polymer nanocarriers are still in preliminary stages, *in vitro* drug release behavior and cytotoxicity data are available. These systems are slow in clinical trials because of defects like polymer-related toxicity and low conjugate bioactivity. In the future, more efforts are needed to develop methods for combinations with other stimuli like redox or temperature for specific targeted release.

### Redox-responsive polymers

**Figure 4:**
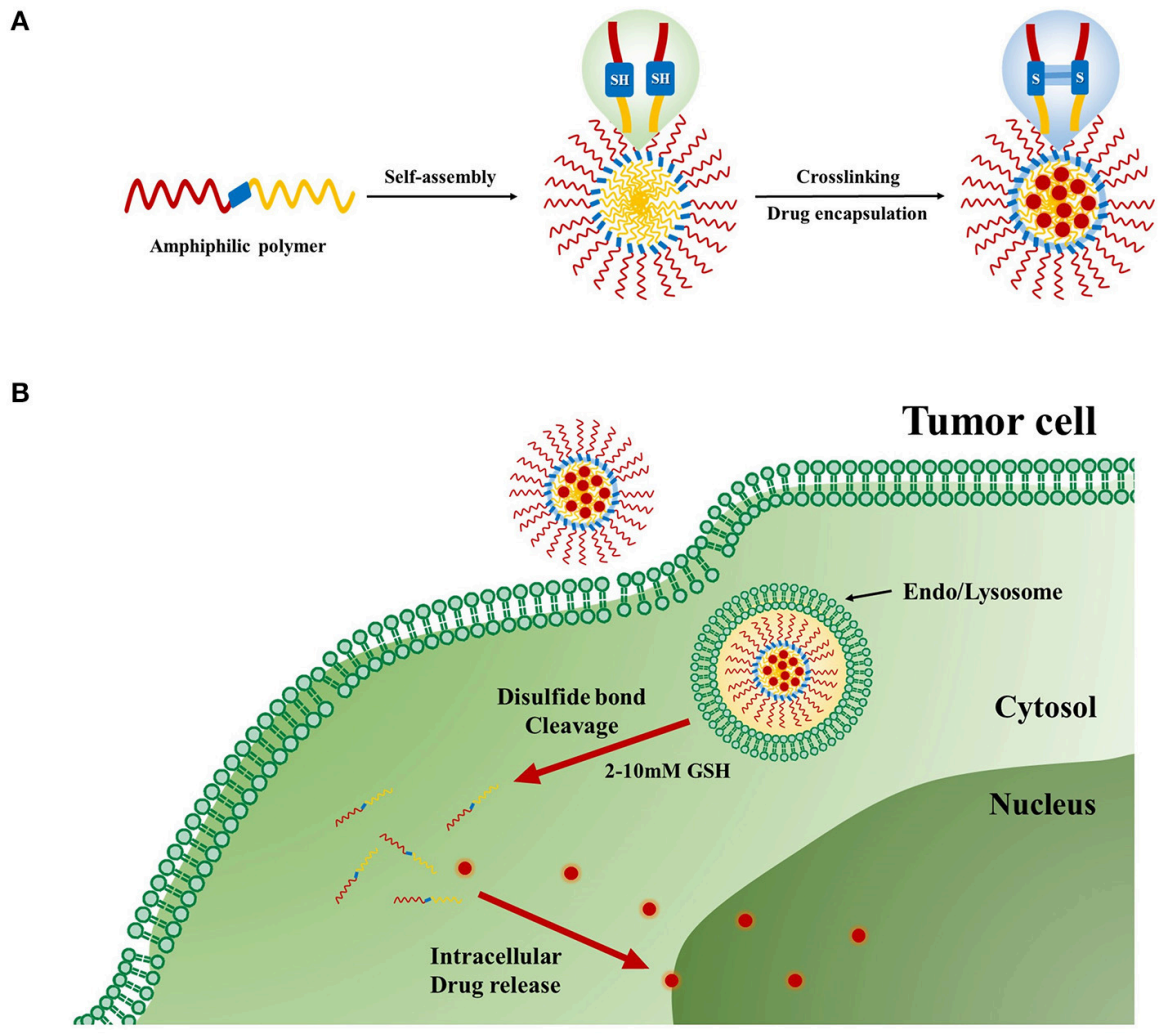
Redox-responsive polymer nanoparticles for tumor-targeted drug Delivery. **(A)** Cartoon representation of drug loaded cross-linked polymer nanoparticles. **(B)** Internalization of the nanoparticles and subsequent intracellular GSH-responsive drug release behavior.

Redox-responsive stimuli are most important for disease therapy and are widely used in polymer drug delivery systems. The disulfide linker is reduction-sensitive and readily cleaved by a high concentration of GSH. GSH, a tri-peptide consisting of glutamate, cysteine, and glycine, is an abundant thiol species in the cytoplasm. GSH is a major reducing ligand in biochemical processes (Thambi et al., 2016a). GSH/glutathione disulfide (GSSG) is most abundant in the cytoplasm (1–10 μM), whereas the concentration drops to about 2–20 μM outside of cells. Moreover, tumor tissues showed at least 4-fold higher GSH concentration than normal tissue (Jones et al., 1998; Choi et al., 2012). The GSH level is related to many human diseases like neurodegenerative diseases, liver diseases, stroke, seizure, and diabetes (Estrela et al., 2006). The difference in the redox environment has been used for developing redox-responsive drug delivery systems (Schafer and Buettner, 2001) (Figure 4). Many research groups have attempted to prepare self-assembled amphiphilic copolymers via disulfide-containing crosslinkers, oxidization of thiol groups, and disulfide-thiol exchange reaction. Generally, there are two approaches to use of disulfide bonds in polymer systems. One is the alteration of disulfide bonds on the backbone chains of the polymer. The other is to use GSH-sensitive crosslinking agents incorporated either in the shell or the core of micelles. These polymeric nanocarriers are very stable in blood circulation but are internalized by cells once they accumulate in targeted sites (Estrela et al., 2006; Li-Ping et al., 2010). These polymeric nanoparticles (PNPs) disintegrate and release their cargo. Due to advantages of PNPs, such as biocompatibility, water solubility encapsulation of hydrophobic drugs into copolymers has been explored for cancer therapy. Because of their amphiphilicity, copolymers can be self-assembled into different nanostructures with different ratios of hydrophobic and hydrophilic groups. By incorporating linkages such as disulfide bonds into these copolymers, redox-responsive polymeric nanocarriers can be prepared (Zhang et al., 2017).

**Figure 5:**
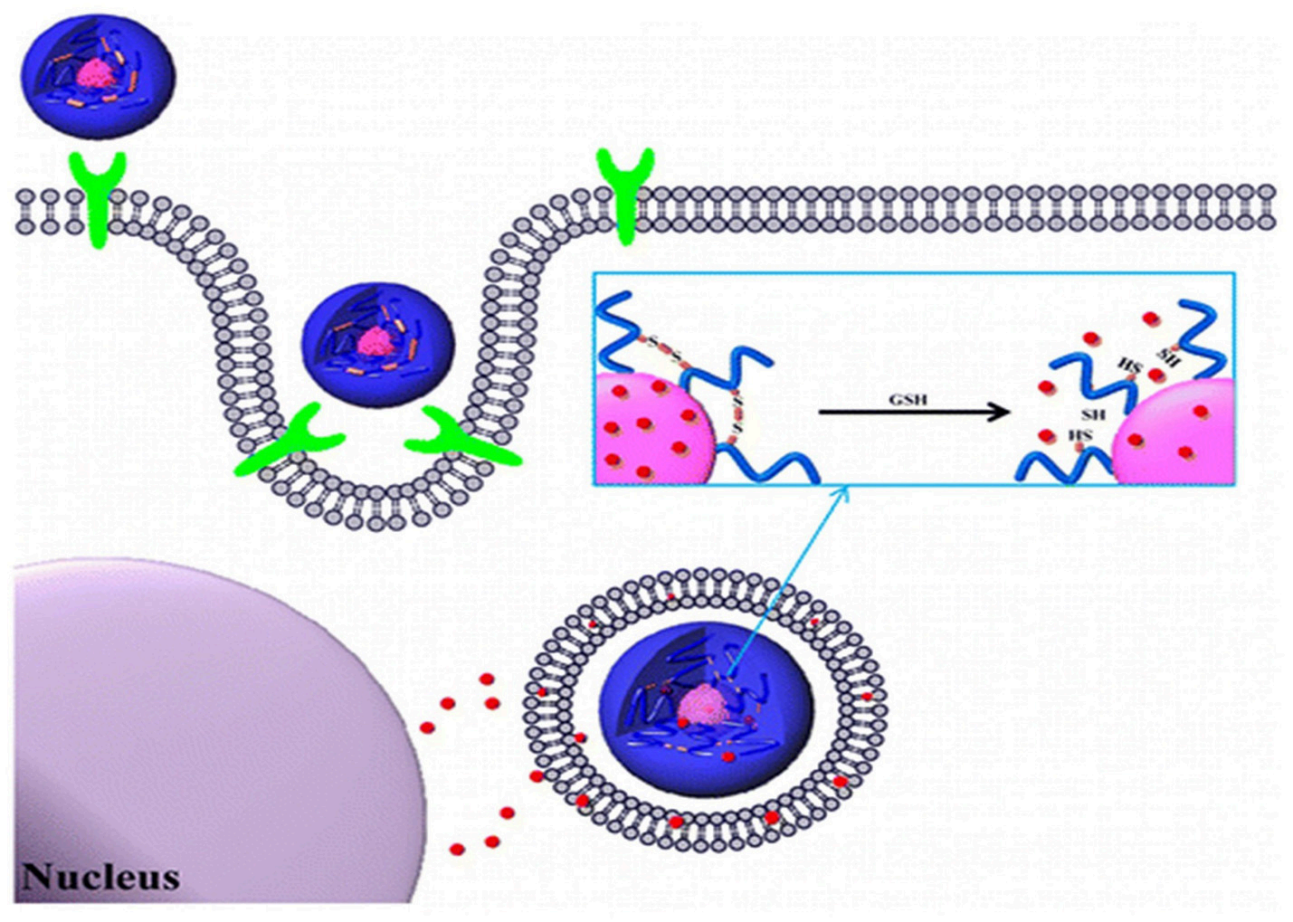
Bioreducible shell-cross-linked hyaluronic acid nanoparticles for tumor-targeted drug delivery reprinted from Han et al. (2015) with the permission of ACS publications.

Recently, Han et al. reported redox-responsive tumor-targeted PNPs based on hyaluronic acid (HA)-polycaprolactone (PCL) block copolymer (Han et al., 2015). The HA shell was cross-linked via disulfide linkage (Han et al., 2015). Doxorubicin (DOX) was efficiently loaded into nanoparticles with high drug loading efficiency. The DOX-loaded HA nanoparticles retarded premature drug release under physiological conditions (pH 7.4), whereas the drug release rate was increased in the presence of GSH bonds in the cytoplasm. The tumor-targeting and therapeutic efficacy of HA-PCL copolymers with disulfide linkages was significantly higher than that of non-cross-linked nanoparticles and free chemotherapeutic drugs (Figure 5). In another report, Han et al. developed cross-linked HA polymer nanocarriers via disulfide bond formation. DOX was physically loaded in the HA NPs (Han et al., 2015). The copolymer exhibited enhanced *in vivo* tumor targetability and therapeutic efficacy compared to non-cross-linked HAMs. Interestingly, this polymer demonstrated improved pharmacokinetics and tumor accumulation, and it also exhibited excellent therapeutic efficacy. In conclusion, this polymer can be used for targeted cancer therapy.

The same group also attempted to prepare carboxymethyl dextran (CMD) with lithocholic acid (LCA) through a disulfide linkage (Thambi et al., 2014a). DOX was loaded into the polymer nanoparticles with 70% loading efficacy. Polymeric NPs released DOX in the presence of 10 mm GSH. Moreover, drug release was significantly retarded in physiological buffer (pH 7.4). DOX-loaded polymer NPs showed greater toxicity to SCC7 cancer cells than DOX-loaded nanoparticles without disulfide bonds. Confocal laser scanning microscopy confirmed that nanoparticles could efficiently deliver DOX into the nuclei of SCC7 cells. An *in vivo* bio-distribution study indicated that DOX selectively accumulated at tumor sites after systemic administration into tumor-bearing mice. Overall, it is apparent that newly designed polymer NPs can be used for cancer therapy. The same group recently reported amphiphilic diblock copolymer bearing disulfide linker consisting of PEG and poly(γ-benzyl L-glutamate). Due to its amphiphilic nature, the copolymer formed PNPs in aqueous conditions. DOX was loaded into PNPs with 75% loading efficacy. The micelle released DOX at 10 mM GSH, mimicking intracellular conditions. *In vitro* data revealed that DOX-loaded reduction-sensitive micelles were toxic to SCC7 cells compared to reduction-insensitive control micelles.

Xu et al. recently reported GSH-responsive poly(ethylene glycol)-*b*-polycarbonate-*b*-poly(ethylene glycol) triblock copolymer as a DOX drug carrier (Xu et al., 2015). This nanocarrier showed glutathione-triggered drug release and cytotoxicity to cancer cells. The triblock copolymer exhibited promising anticancer drug carrier for potential clinical applications. In another report, Wen et al. reported a novel, disulfide-containing prodrug of CPT to prepare self-assembling nano micelles (CPT-SS-PEG-SS-CPT) with a redox-sensitive drug release mechanism (Wen, 2014). Under tumor-relevant reductive conditions, GSH-mediated CPT release was observed by reductive cleavage of the disulfide linker. More interestingly, cell proliferation assays demonstrated the pharmacological efficacy of CPT released from micelles under tumor-relevant GSH concentrations. In another report, Nam et al. recently prepared paclitaxel-conjugated polymeric micelle consisting of poly(ethylene glycol) and arginine-grafted bioreducible poly(disulfide amine) (Nam et al., 2012). This polymer exhibited high release of paclitaxel in the cytoplasm. Overall, *in vitro* and *in vivo* results indicate that micelle-based delivery systems are well-suited for drug delivery. Even though exciting research is occurring in the area of redox stimuli-responsive polymer nanocarriers, it is difficult to achieve specific redox molecular mechanism-based controllability due to the complex biological environment and heterogeneity of tumor cells.

### Hypoxia-responsive polymers

**Figure 6:**
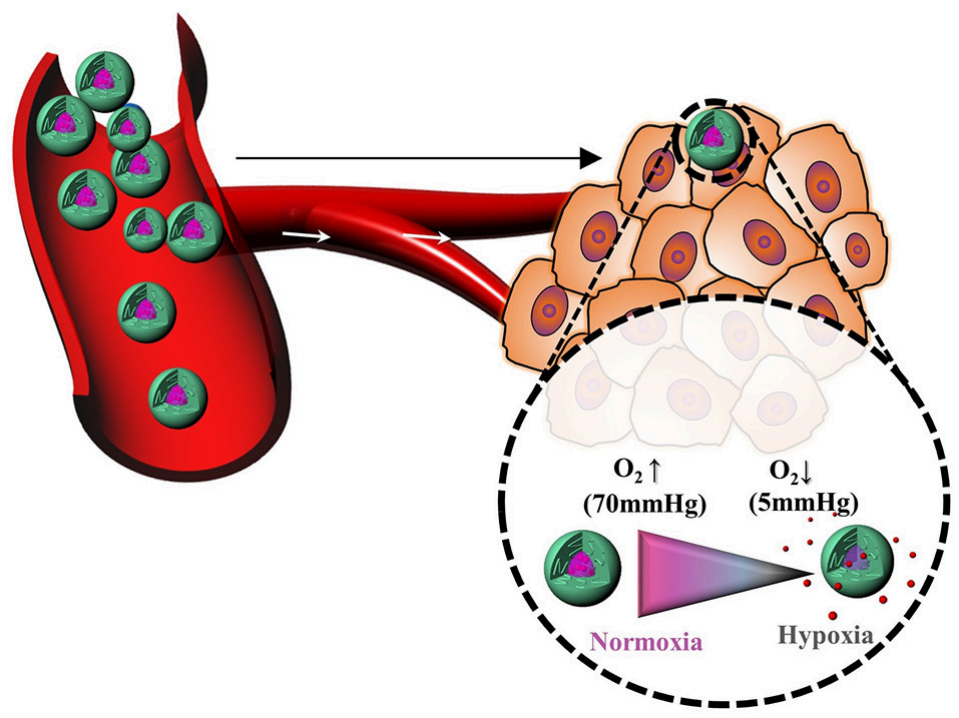
Cartoon representation of hypoxia-responsive polymers for drug delivery.

Hypoxia, a pathological state of inadequate oxygen, is involved in the pathogenesis of intractable diseases such as cancer, cardiopathy, ischemia, rheumatoid arthritis, and vascular diseases (Harris, 2002; Cabane et al., 2012; Thambi et al., 2014b). Oxygen partial pressure decreases from the surface to the interior of tumors, reaching as low as 0–5 mmHg in some regions. Hypoxia also plays a substantial role in resistance to chemotherapy in cancer patients. Hypoxic and normoxic cells have remarkably different microenvironments, providing an opportunity for tumor-specific drug delivery with reduced oxygen partial pressure as the trigger (Brown and Wilson, 2004). However, hypoxia-responsive polymeric drug carriers have been less explored (Figure 6). In this section, we discuss recent progress in hypoxia-responsive nanocarriers for cancer imaging and therapy. In an attempt to develop the nanocarrier for cancer therapy, the azo linker-incorporated amphiphilic polymer consisting of carboxymethyl dextran—black hole quencher 3 was synthesized for targeted delivery to cancer (Son et al., 2018). The polymer conjugate could self-assembled into nanoparticles under aqueous conditions. The DOX, loaded polymer nanoparticles released DOX remarkably under hypoxic conditions because the azo bonds in BHQ3 are reduced under hypoxic conditions. These polymer nanoparticles exhibited oxygen-dependent intracellular release of DOX under hypoxic conditions. When polymer nanoparticles were systemically injected into the tumor-bearing mice, the most significant quantity of NPs was found in tumor tissue.

Thambi et al. reported PNPs that can selectively release DOX under hypoxic conditions (Thambi et al., 2014b). To prepare hypoxia-responsive PNPs, a 2-nitroimidazole derivative was conjugated to the backbone of CMD. DOX was encapsulated into PNPs. Interestingly, DOX was released slowly under normoxic conditions. However, the drug release rate increased under hypoxic conditions. *In vitro* and *in vivo* experiments indicated high tumor accumulation and anti-tumor efficacy. In another study, the same group reported hypoxia-sensitive block copolymer composed of PEG and poly(ε-(4-nitro)benzyloxycarbonyl-l-lysine) (Thambi et al., 2016b). Due to its amphiphilic nature, the block copolymer formed micelles, and DOX was loaded in aqueous conditions. Interestingly, the DOX-loaded micelles exhibited intracellular release of DOX under hypoxic conditions, indicating a high potential for the use of hypoxia-sensitive micelles as drug carriers for cancer therapy. In another report, azo-based hypoxia-responsive prodrug micelles consisting of PEG-hexanethiol (PEG-C6) with combretastatin A-4 (CA4) were prepared (Liu et al., 2015). This polymer conjugates self-assembled into micelles, which can encapsulate the anticancer drug DOX.

The drug release behavior of DOX and free CA4 was observed under hypoxic conditions. The drug release data indicated that PEG-C6-azo-CA4 micelles are a promising candidate for cancer treatment. In another report, Kulkarni et al. reported that diblock copolymers consisting of poly(lactic acid)–azobenzene–poly(ethylene glycol) can self-assemble to form polymersomes in an aqueous medium (Kulkarni et al., 2016). The anticancer drugs gemcitabine and erlotinib were loaded into polymersomes. These polymersomes released the encapsulated drugs to hypoxic pancreatic cancer cells, and reduced cell viability was subsequently observed in both monolayer and spheroidal cultures. Perche et al. reported hypoxia-induced siRNA delivery using a polymer nanocarrier consisting of PEG, azobenzene, polyethyleneimine, and phospholipid (Perche et al., 2014). The nanocarriers create efficient complexes with siRNA in aqueous solutions. *In vitro* and *in vivo* experiments indicate enhanced gene silencing under hypoxic conditions that mimic the hypoxic tumor microenvironment. An ideal hypoxia-responsive nanocarrier could be helpful in treating cancer and intractable diseases such as ischemic stroke, cardiopathy, and rheumatoid arthritis.

### ROS-responsive polymers

By utilizing the high accumulation of reactive oxygen species (ROS) in some disease tissues, ROS-response polymer nanoparticles are also an effective mechanism to control targeted drug release. It has been reported the mucosal ROS concentrations in inflammatory tissues and colon cancer were 10- to 100-fold higher than that of normal tissues. In this section, we will discuss recent progress in reactive oxygen species (ROS)-responsive carrier systems. ROS refers to a class of oxygen-derived chemical species produced by the body. Typical ROS species include hydrogen peroxide (H_2_O_2_), singlet oxygen (^1^O_2_), superoxide (O${}_{2}^{-}$), and hydroxyl radicals (HO•). ROS can transform from one to another via a cascade of reactions (MacKay and Knock, 2015; Panieri and Santoro, 2015). The primary sources of endogenous ROS generation are mitochondria, endoplasmic reticulum, and NADPH oxidase (Burgoyne et al., 2013). Produced ROS are critical for the production of several hormones, regulation of cell signaling, and mediation of inflammation. An excess of ROS can increase the risk of mutated cellular DNA, which is closely associated with progression of several cancer cell types (Trachootham et al., 2009). Though many stimuli-responsive PNPs have been extensively explored in cancer therapy, the incorporation of ROS-responsive PNPs for drug release has gained significant attention recently, and increasing evidence suggests that several pathogenic processes involve elevated ROS (Lee et al., 2013). In the literature, inorganic nanoparticles were studied with ROS-responsive moieties for biomedical applications such as cancer-targeted drug delivery systems and cell therapy platforms for inflammation-related diseases (Saravanakumar et al., 2017). Suk Shim et al. developed a ROS-cleavable thioketal-based cationic polymer with a cancer-targeting peptide that led to selective and targeted gene delivery in cancer cells (Suk Shim and Xia, 2013). Cleavage of thioketal linkers under ROS conditions led to the intracellular release of complexed DNA in human prostate cancer cells. That study showed that high levels of intracellular ROS in cancer cells act as biological stimuli that can be used for gene delivery in cancer cells. Another early report described a new diblock copolymer consisting of PEG and poly(lactic-co-glycolic acid) with an ROS-cleavable thioketal linker (Li et al., 2016). The anticancer drug DOX was loaded in PNPs, and DOX-loaded NPs escaped from endosomes and entered the cytoplasm. Thioketal-containing linker was cleaved in the presence of intracellular ROS, and the loaded DOX was rapidly released to induce apoptosis of Cal27 cells. Thioether linker was also used to design ROS-responsive stimuli-responsive PNPs. In a recent study, PEG and poly(propylene sulfide) block copolymer was synthesized (Napoli et al., 2004) and self-assembled into PNPs. Interestingly, an ROS oxidative environment led to an abrupt hydrophobic-to-hydrophilic transition, destabilization, and eventual disruption. This material is beneficial for oxidation-related diseases such as inflammation and cancer. Similar to the sulfide groups present in thioether-containing polymers, selenium-containing polymers, and tellurium-containing polymers have been developed for ROS-responsive stimuli-responsive PNPs. In a recent report, Deepagan et al. reported diselenide-crosslinked ROS-responsive PNPs (Deepagan et al., 2016). The triblock copolymer was synthesized using PEG and polypeptide derivatives consisting of diselenide groups. During micelle formation, DOX was loaded into PNPs. Tumor regions of ROS abundance triggered rapid release of DOX from the copolymer and effectively suppressed tumor growth. This polymer exhibited maximal therapeutic efficacy.

## Exogenous stimuli-responsive polymers

### Light-triggered polymers

**Figure 7:**
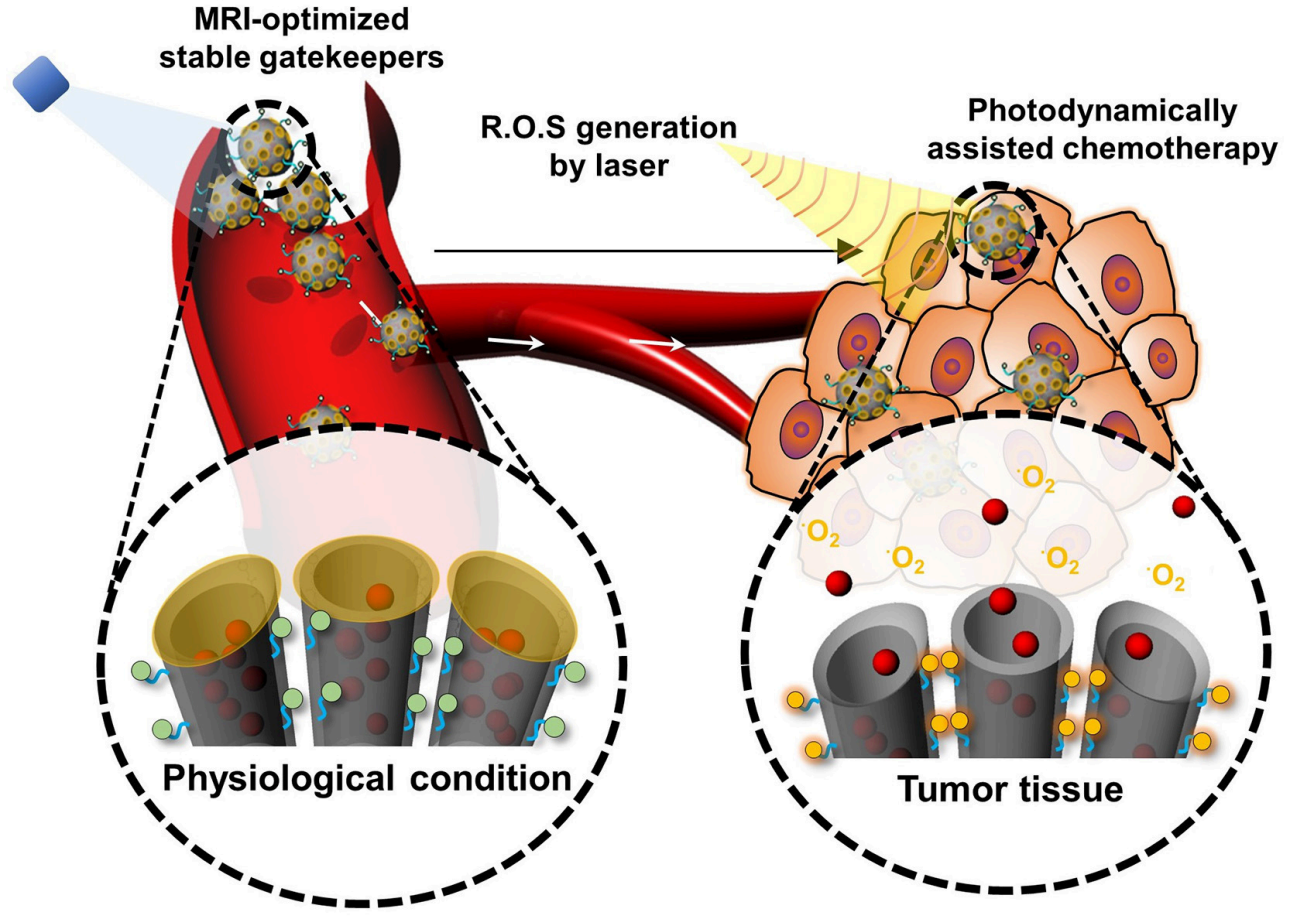
Schematic illustration of the photodynamically assisted chemotherapy. At the target site, the high level of ROS cleaves the ROS sensitive linker resulting in triggering drug release.

Tumors with high interstitial pressure and a dense extracellular matrix affect the uptake of nanoparticles and tumor penetration. Photothermal therapy (PTT) has overcome these barriers for hyperthermia damage to cancer cells, enhanced tumor penetration of nanoparticles, and triggered release of cargo (Haijun et al., 2015). The photothermal effect is owing to their strong ability to absorb near-infrared (NIR) energy and transform it into heat for tumor ablation and deep penetration to achieve on-demand release in drug-resistant malignant cells (Dongdong et al., 2016). However, the light penetration depth is still an obstacle restraining their application for deep tissues. Photodynamic therapy (PDT) is one kind of minimally invasive treatment that combines light at appropriate wavelengths with a photosensitizer (photoactive drug) to destroy target cells by producing highly toxic ROS. PDT, which uses a photosensitizer to induce ROS-mediated cell death, is a potent therapeutics for tumor-targeted therapy (Lovell et al., 2010; V R et al., 2018). Interestingly, many studies have shown that the photodynbamic effect can improve the therapeutic effect of nanoparticle-mediated chemotherapy. After entering a cell by endosomal uptake, if the nanoparticles are irradiated with light to cause the photodynamic effect, the endosomal membrane is oxidized by generated ROS and disrupted, a phenomenon known as photochemical internalization (PCI). PCI is now a well-established process and has been applied to enhance the delivery of various therapeutic molecules. Because the delivered drug is degraded in the endosomal environment, a system is likely to take advantage of PCI to assist with successful intracellular drug delivery. So, the combination of both PS and ^1^O_2_-cleavable linker could be a great strategy to prepare a photodynamically controllable drug delivery platform. Meanwhile, to control drug release, ^1^O_2_ is generated, which can not only trigger drug release, but additionally alleviates endosomal escape. Furthermore, a dual effect of PDT by ^1^O_2_ and chemotherapy through released chemotherapeutics can be anticipated as above. Thus, the development of a photodynamically controlled drug delivery system will undoubtedly be promising in an advanced photo-responsive platform. Our group recently designed a biostable ROS-responsive drug delivery system (V R et al., 2018). Mesoporous silica nanoparticles (MSNPs) are conjugated with DOTA chelates via an ROS sensitive aminoacrylate (AA) linker. The chlorin e6-conjugated-poly (ethylene glycol) copolymer is further conjugated to MSNPs. The DOTA groups presented on the MSN are used for *in situ* gadolinium (III) cross-linking (RG-MSN). This drug delivery system facilitates the monitoring of the bio-distribution of the drug carrier by magnetic resonance imaging (Figure 7). The anticancer drug DOX is loaded into the pores of MSNs for intracellular drug delivery using a dialysis method to produce DOX-RG-MSN. The DOTA groups presented on the RG-MSN are used for *in situ* gadolinium (III) cross-linking to act as a gatekeeper. Tumor growth can be effectively inhibited after treatment with DOX-RG-MSN in combination with PDT-assisted chemotherapy. ^1^O_2_ is created upon irradiation with light and is utilized for photodynamically triggered drug release. Using this ^1^O_2_-responsive nanocarrier delivery system, DOX can easily reach the tumor site and be accumulated in the nuclei to kill the cancer cells, therefore decreasing the side effects of chemotherapy.

### Temperature-responsive polymers

Temperature is a vital factor in controlled drug release. In general, tumor tissues have higher temperatures than normal tissues (Danhier et al., 2010). By taking advantage of the temperature difference between cancer tissues and normal tissues, stimuli-responsive polymer nanoparticles can be triggered to enhance their drug release in tumors (Shi et al., 2013a,b). An alternative temperature-responsive strategy is that the tumor site could be heated by external triggers (US, magnetic field, etc.) (Zhao et al., 2011) to improve the drug release within the tumor vasculature microenvironment. In general, thermo-sensitive nanocarriers are designed to retain their payloads around the physiological temperature of 37°C and release the payloads rapidly when the temperature is increased higher than 40–45°C. Temperature-responsive polymers have received considerable attention because of the phase transition induced by the alternation of the external environment. Various polymers have been shown to exhibit critical solubility temperature (CST) (Liu et al., 2009), and poly-N-isopropylacrylamide (PNIPAAm) is often used due to its lower critical solution temperature (LCST) of 32°C. The polymer side-chain isopropyl groups can be easily hydrated or dehydrated to induce a reversible variety of hydrophilicity or hydrophobicity (Alvarez-Lorenzo and Concheiro, 2014). With thermo-responsive nanoplatforms, the present challenge is to maintain the safety of the platforms without compromising their sensitivity to minor temperature changes (Mura et al., 2013).

### Difficulties for stimuli-responsive polymer in potential clinical applications

In addition to the above mentioned stimuli-responsive polymer nanoparticles for glucose, electro-responsive systems have been explored to control drug release (Murdan, 2003; Guo et al., 2015). The combination of dual stimuli-responsive polymer nanoparticles such as thermo- and pH-responsive systems (Guo et al., 2015), thermo- and light-responsive systems (Murdan, 2003) redox- and pH-responsive systems (Pan et al., 2012), and ultrasonic and magnetic responsive systems have been explored (Fang et al., 2010). In the past decades, many stimuli-responsive polymer nanoparticles have been developed, though all have limitations for drug delivery (Crommelin and Florence, 2013; Lee et al., 2015). However, Visudyne, for PDT, using a stimuli-responsive nanoplatform concept has been approved by the Food and Drug Administration (FDA). However, other stimuli-responsive polymers are still in the clinical stage, as shown in Table 2 (Shaffer et al., 2007; Lindner et al., 2008; Schwartz et al., 2009; Rivera Gil et al., 2010; Etheridge et al., 2013). Here, we highlighted the key points for successful drug carrier translation in these smart carriers. Even though there are a remarkable number of publications about stimuli-responsive polymers, little has been published on the relevance of safety and efficacy of smart polymer nanocarriers in animals to be predictive of clinical effects in humans (Crommelin and Florence, 2013). Animal models such as mouse and rat are tools for understanding the molecular basis and pathology of diseases. However, current animal models cannot accurately reproduce human diseases because of inbreeding, limiting application to diverse human beings. The major issues for clinical translation of nanomedicine include a series of complex biological barriers to nanocarriers, including the safety of the nanomaterials, their biodegradability. Thirdly, significant issues for clinical translation of nanomedicine include nanoparticle preparation needs to be simplified, industrial scale-up validation, and batch-to-batch reproducibility.

**Table 2:** Polymeric micelles systems in clinical trials.

**Name of the product**	**Polymer structure/Identified**	**Therapeutics**	**Application**	**Phase**	**Company name**
NK911	PEG-*b*-p(Asp-DOX)	Doxorubicin	Pancreatic and colorectal cancer	II	Nippon Kayaku, Japan
NK105	PEG-*b*-p (ASP-4-phenyl-1-butanol)	Paclitaxel	Stomach, breast cancer	III	NanoCarrier/Nippon Kayaku, Japan
NC-6004	PEG-*b*-p(Glu)	Cisplatin	Pancreatic, head and neck, lung, bladder and bile duct cancer	III	NanoCarrier/Nippon Kayaku, Japan
NK012	PEG-*b*-p(Glu-SN-38)	SN-38	Breast, lung, colorectal cancer	II	Nippon Kayaku, Japan
NC-6300	PEG-*b*-p(Asp-hydrazone)	Epirubicin	Solid tumors	I	NanoCarrier, Japan/Kowa
NC-4016	PEG-*b*-p(Glu)	DACH-Platinum	Solid tumors and lymphoma	I	NanoCarrier, Japan

## Conclusion

Various stimuli-responsive polymers have been established to deliver the anticancer drugs in response to a range of endogenous (redox, pH, hypoxia, and ROS) and exogenous stimuli (temperature, and ultrasound). The endogenous stimuli-responsive system relies on the abnormal environments in diseased tissues for target-specific drug delivery, while the exogenous stimuli-responsive one needs prior knowledge on the location of the target site for effective therapy. Because of the heterogeneity in the physiological conditions, exogenous stimuli-responsive drug delivery systems would be more favorable. A number of polymers have been explored for preparation of different stimuli-responsive drug delivery systems. Nonetheless, a number of challenges remain and future studies will need to address several issues.

First, the biocompatibility and biodegradability of the polymers must be improved. Second, it is essential to understand how stimuli-responsive polymers interact with the biological components. Upon administration, stimuli-responsive polymers encounter a variety of biological molecules, cells, and tissues. The surface of stimuli-responsive polymers would be quickly covered with biological molecules such as serum proteins which play a vital role in determining the subsequent bio-distribution and cellular responses of polymers. Next, to protect their cargo molecules from biological degradation and lower harmful side-effects in healthy cells and tissues, the polymeric nanocarriers must have the ability to access the target cells while passing the biological barriers and escaping from the reticuloendothelial system (RES). The polymeric nanocarriers need to escape from the RES to improve their circulation time, which is the significant parameter to accomplish efficient delivery of cargo molecules to the target site. For this purpose, along with surface architecture, the various parameters (i.e., shape, size, and surface charge of polymeric nanocarriers) should be tuned. The further understanding of cancer biology and polymer chemistry will catalyze optimization pathways to attain more efficient anti-tumor systems. Additionally, the good permeability of tumor vasculature is mostly constructed in experimental animal models. However, in practice, the growth rate of tumors in the human body is relatively slower than that in animal models, leading to an unsatisfied EPR effect. As a result, it is urgent to improve the progression of active targeting to tumors via ligand-mediated or exogenous stimulus. Getting insights into the differences between normal and pathological tissues will also result in the development of new selective triggers, targeted therapies and a highly promising role for stimuli-responsive nano-assemblies. The complicated synthesis and difficulties in the scale-up of stimuli-responsive polymers are likely to hinder their clinical translation. This is one of the significant reasons why many stimuli-responsive polymers have been reported, whereas only a few of them could enter the clinical stage.

Future opportunities for nanomedicines are looking toward combinations of different types of stimuli to develop multifunctional drug delivery nanosystems. The combing diagnostic and therapeutic agents may be introduced to enable the visual tracking of cancer therapy. In the near future, we anticipate that multifunctional drug delivery nanosystems for cancer therapy will be developed for actual clinical applications.

## Author contributions

NR, HK, JL, and JP wrote and edited the paper.

### Conflict of interest statement

The authors declare that the research was conducted in the absence of any commercial or financial relationships that could be construed as a potential conflict of interest.
